# High Fidelity Simulation For Nurse Training Reduces Unplanned Interruption of Continuous Veno-Venous Hemofiltration Sessions in Critically Ill Patients. a Randomised Controlled Study

**DOI:** 10.1186/2197-425X-3-S1-A12

**Published:** 2015-10-01

**Authors:** S Lasocki, P Lemarié, S Vidal-Husser, S Gergaud, X Verger, J Berton, JC Granry

**Affiliations:** LUNAM Université d'Angers, CHU d'Angers, Angers, France

## Introduction

Continuous veno-venous hemofiltration (CVVH) is often not continuous. Indeed, premature unplanned interruptions of sessions (UI) appear frequently. Nurses are directly implicated in the management of CVVH generators. High fidelity simulation (Sim) is an efficient method for health care provider training, mainly to improve procedural and knowledge skills. Taking advantage of the introduction of CVVH in our ICU as a new technic, we wanted to evaluate Sim for nurses CVVH training.

## Objectives

To assess if Simulation training of nurses reduces unplanned interruption of CVVH sessions.

## Methods

Prospective randomized, single-center, 2-phases study:

(1) Formation, the 30 nurses of our ICU (nurse to patient ratio 1/2.5) received 6 hours of formation for CVVH management (done by Fresenius, France), they were then randomized (with stratification on previous experience with haemodialysis) to receive or not a Sim formation (3x2h sessions). CVVH knowledge was evaluate using 40 questions test.

(2) Evaluation, all the CVVH sessions performed in the ICU were randomized to be managed either by Sim or control nurses (i.e. the nurse in charge of the patient may be changed according to randomization). a minimization equation was used for randomization (including shock, counter-indication to heparin, agitation, prior UI and catheter site). Need for help was prospectively collected. Efficiency of CVVH session was assessed by urea reduction fraction. Rate of UI (defined as a session interruption not prescribed more than 4 hours before) was compared between the 2 groups. Logistic regression was used to evaluate Factors of UI (including SOFA score, Shock (yes/no), anticoagulation (yes/no), delay from admission, agitation, need for help, position of catheter (jugular/femoral). Data (mean ± SD, median[Q1-Q3] or n(%)) were compared using Wilcoxon or Fischer exact test as appropriated.

(SimHeR study, NCT02379234).

## Results

**Figure 1:**
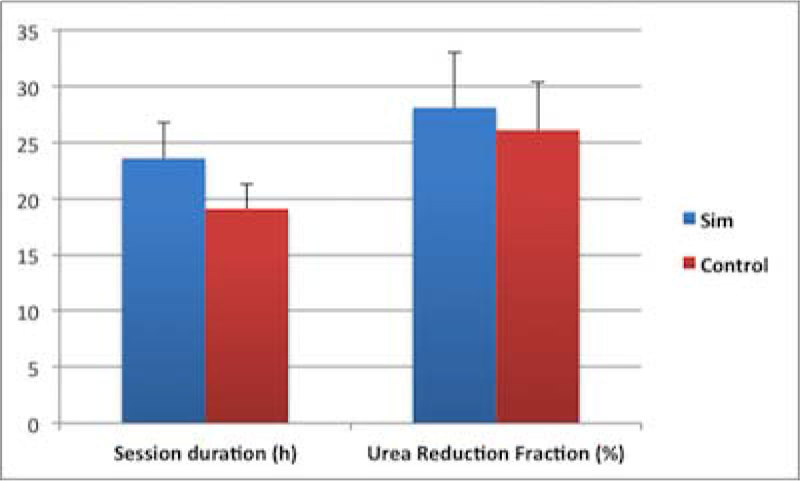
Figure 1

Nurse experience was comparable between the two groups (5[5-7] vs 5[3.5-8] yrs of ICU experience for Sim and C, p = 0.73). Sim nurses had better knowledge scores (14[10.5-15] vs 11[10-12] /20, p = 0.044). During a 13 months period, 106 sessions were randomized (n = 53/group) among 50 patients (age 70 ± 13 yrs, SAPS II 74 ± 23, SOFA 11,5 ± 3,4). Catheter position, absence of anticoagulation, schock, UI during previous session, patient agitation were not different in the 2 groups. Duration and urea reduction were not significantly greater in Sim (figure 1). There were significantly less UI in Sim group (27(62%) vs 38(88%) UI, p < 0.009) and Sim nurses needed less frequently help (0[0-1] vs 3[1-4] times/session, p < 0.0001). The 2 factors associated with UI in multiple regression analysis were session SOFA score (p = 0.048) and study group (p = 0.03).

## Conclusions

CVVH nurse training using simulation is associated with a reduction in unplanned interruption of CVVH session and less need for help. Simulation may offer new perspective for nurse training in the ICU.

